# Cognitive mechanisms underlying instructed choice exploration of small city maps

**DOI:** 10.3389/fnins.2015.00060

**Published:** 2015-03-20

**Authors:** Sofia Sakellaridi, Peka Christova, Vassilios N. Christopoulos, Alice Vialard, John Peponis, Apostolos P. Georgopoulos

**Affiliations:** ^1^Center for Cognitive Sciences, University of MinnesotaMinneapolis, MN, USA; ^2^Brain Sciences Center, Veterans Affairs Medical CenterMinneapolis, MN, USA; ^3^Department of Neuroscience, University of Minnesota Medical SchoolMinneapolis, MN, USA; ^4^Division of Biology and Biological Engineering, California Institute of TechnologyPasadena, CA, USA; ^5^School of Architecture, College of Architecture, Georgia Institute of TechnologyAtlanta, GA, USA

**Keywords:** instructed choice exploration, spatial decision making, eye fixations, map reading

## Abstract

We investigated the cognitive mechanisms underlying the exploration and decision-making in realistic and novel environments. Twelve human subjects were shown small circular U.S. city maps with two locations highlighted on the circumference, as possible choices for a post office (“targets”). At the beginning of a trial, subjects fixated a spot at the center of the map and ultimately chose one of the two locations. A space syntax analysis of the map paths (from the center to each target) revealed that the chosen location was associated with the less convoluted path, as if subjects navigated mentally the paths in an “ant's way,” i.e., by staying within street boundaries, and ultimately choosing the target that could be reached from the center in the shortest way, and the fewest turns and intersections. The subjects' strategy for map exploration and decision making was investigated by monitoring eye position during the task. This revealed a restricted exploration of the map delimited by the location of the two alternative options and the center of the map. Specifically, subjects explored the areas around the two target options by repeatedly looking at them before deciding which one to choose, presumably implementing an evaluation and decision-making process. The ultimate selection of a specific target was significantly associated with the time spent exploring the area around that target. Finally, an analysis of the sequence of eye fixations revealed that subjects tended to look systematically toward the target ultimately chosen even from the beginning of the trial. This finding indicates an early cognitive selection bias for the ensuing decision process.

## Introduction

To make good decisions within a novel environment, we first have to explore it. But how people explore novel environments to make decisions is poorly understood. Consider a hypothetical scenario that you have been accepted by a graduate school and you are visiting the university for the first time to find a house to rent. The school has provided you with a map that marks the student houses around campus and also gives you information about the bus stations, classrooms, libraries, food service, etc. Abstractly, you face an example of a common decision problem, in which you have to explore and evaluate all the alternative options to find the best place to rent. Choosing between alternative options requires assigning and integrating values along a multitude of dimensions (e.g., rental rate, amenities, distance from school, etc.). How do people explore novel environments to extract information and make decisions is considered one of the fundamental problems in decision science.

After many years of intense research in various disciplines ranging from psychology to economics, substantial progress has been made in understanding the cognitive mechanisms of decision-making in a variety of tasks. A series of experimental studies in humans and animals have provided evidence that the brain makes simple decisions by integrating various relevant determinants of an option into a single subjective value, and then comparing these values to make a choice (Roesch and Olson, 2004; Padoa-Schioppa and Assad, 2006; Padoa-Schioppa, 2007, 2011; Wallis, 2007; Rangel and Clithero, 2013). Although these studies have contributed significantly in understanding the cognitive mechanisms of decision making, they have focused heavily on simple decisions that (a) take place in artificial environments, and (b) rely on values of the alternative options that depend only on the options themselves and not on the environmental properties.

Whereas in many decisions the environmental properties do not influence the economic values of the alternatives, such as deciding between products in a grocery store, there are other cases in which the value of an option strongly depends on its environment. For instance, student houses that are closer to campus are usually more expensive than distant houses, even when they share similar characteristics. Solving this type of decision problem, like any other executive process, requires exploring the surroundings of the alternative options, extracting information about the properties of the environment, and integrate this information with value information related to the options themselves.

In this study, we designed a novel experiment to investigate how people explore realistic environments to make decisions. For that purpose, we used a set of real maps of various U.S. metropolitan cities with different street network types, and marked two locations as possible post offices from which subjects had to pick one as their choice. We then investigated the map attributes and sequential eye fixations to gain an insight into the dynamics of the decision making process and the reasons for the subjects' ultimate choice.

## Materials and methods

### Subjects

Twelve healthy right-handed subjects, 6 women and 6 men, participated in this study as paid volunteers. They ranged in age from 19 to 58 years (women's age 36.8 ± 5.8 years, mean ± SEM; men's age 38.8 ± 7 years). The age did not differ significantly between the two genders (*P* = 0.36, *t*-test). The study protocol was approved by the relevant Institutional Review Boards, and an informed consent was obtained from all the participants prior to the study based on the Declaration of Helsinki.

### Stimuli

Stimuli were 20 circular maps of 1-mile diameter urban areas extracted from street center-line maps representing several U.S. Metropolitan Statistical Areas (Atlanta, GA; Baltimore, MD; Chicago, IL; Los Angeles, CA; New York, NY; Pittsburgh, PA; St. Louis, MO; Tampa, FL; Washington, DC). The sample was chosen to exemplify 5 different street networks types (Southworth and Owens, 1993; Peponis et al., 2007; Christova et al., 2012), namely: (i) *regular grids*, i.e., orthogonally intersecting patterns of streets, (ii) *colliding grids*, i.e., multiple intersecting regular grids rotated with respect to one another, (iii) *curvilinear grids*, i.e., intersecting patterns of curvilinear streets, (iv) *cul-de-sacs*, i.e., hierarchically branching street networks, and (v) *supergrids*, i.e., sparsely spaced orthogonally intersecting main arteries with irregular street patters filling-in the large blocks surrounded by the arteries.

**Figure 1:**
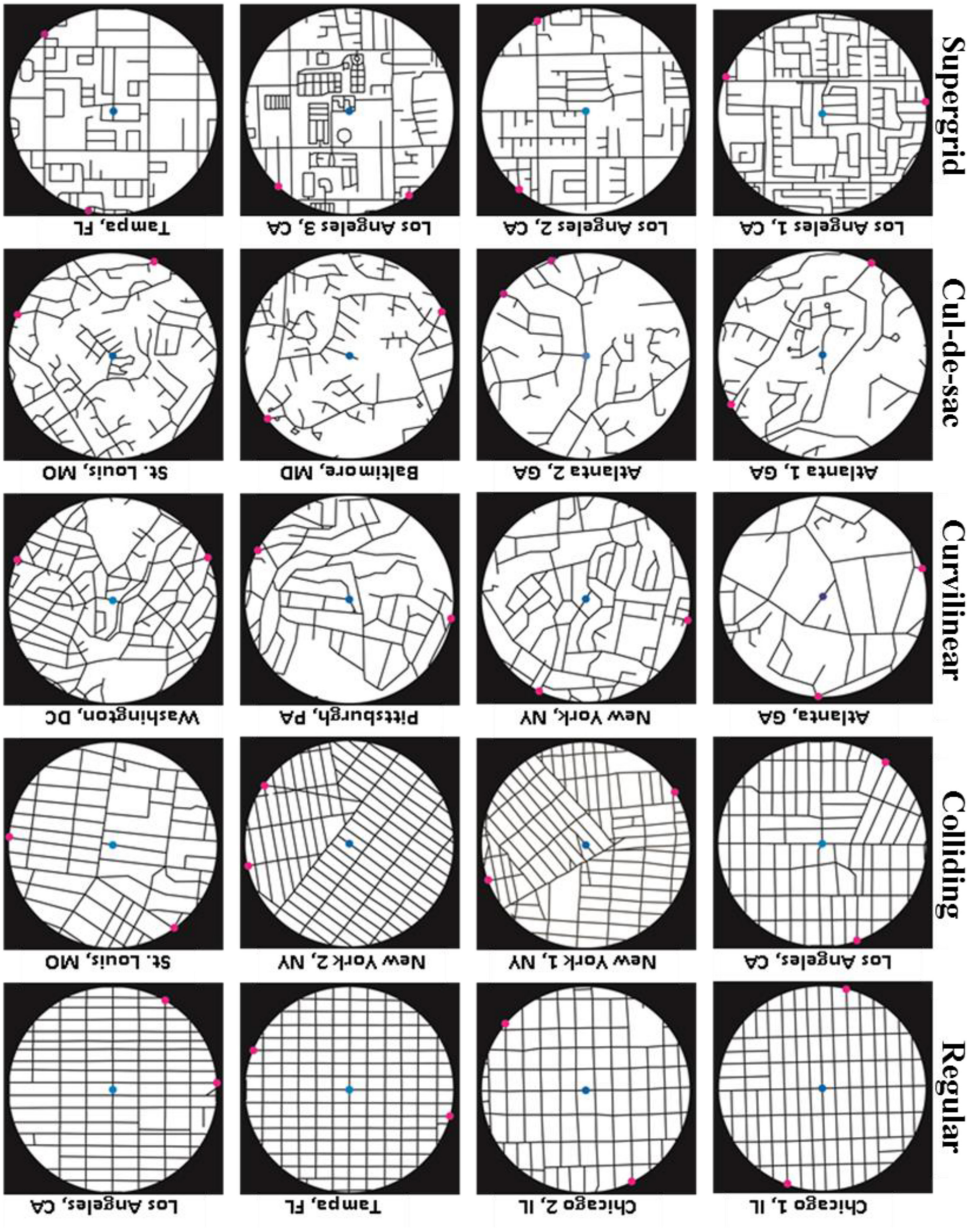
**Map stimuli**. Magenta dots denote the two potential locations (targets) for the post office. Blue dot marks the center of the map (shown here for illustration purposes).

In each map, two targets were marked on the circumference (Figure 1). The locations of the two targets were selected randomly among the points at which the street network intersected the stimulus map perimeter. Four stimuli per street network type (total of 20) were presented to each subject in a pseudorandom sequence.

### Experimental paradigm

#### Task

**Figure 2:**
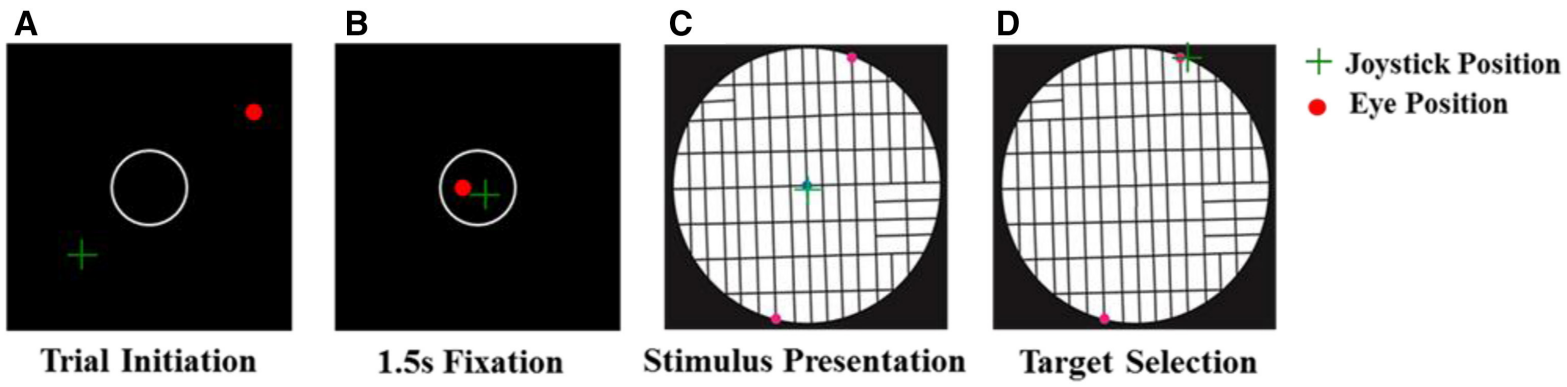
**Task sequence. (A)** Trial starts with the presentation of an open circle on the center of a black screen. **(B)** Subject is required to fixate his/her eyes and place the mouse inside the circle for 1.5 s. **(C)** Stimulus is presented and subject explores the map by moving his/her eyes in order to decide between two alternative positions to post its mail. **(D)** Subject chooses the post office location by clicking the mouse at the desired position.

We used an instructed choice task in which subjects were required to choose one of two locations for a hypothetical post office to post their mail (Figure 2). At the beginning of the trial, an open circle was presented at the center of a black screen. Subjects were instructed to fixate their eyes there and place the mouse cursor inside that circle. After 1.5 s of fixating and holding at the center, the circle was turned off and the stimulus map appeared. The subjects were asked to choose between two alternative post office locations to post their mail by clicking the mouse in the desired location. The subjects were instructed not to trace a path with the mouse cursor, and the experiment proceeded at the subjects' pace. No further instructions were given to the subjects on how to evaluate the alternative options. Subjects sat comfortably on a chair with chin and arm supported to stabilize the head and body. The subject's right forearm manipulating the mouse lay on a firm horizontal support. Stimuli were presented on a computer screen placed at eye level and at a distance of 78 cm in front of the subject.

#### Data acquisition

The experiment was controlled by a program written in Visual basic (Microsoft Visual Basic 2005, version 8.0). Relevant data included the times of presentation of stimuli, the *x*-*y* position of the mouse (sampled at 200 Hz), and the *x*-*y* position of the eyes (sampled at 200 Hz). The eye position was recorded using a video-based pupil/corneal reflection tracing system (model EGL-400, ISCAN, Inc. Burlington, MA).

### Data analysis

#### Spatial analysis of eye positions

Isolines were used to characterize the overall spatial distribution of eye positions during map exploration, as follows. For each map, we superimposed the eye positions of all subjects, and computed the probability of the spatial density of eye position, as follows. First, we fitted a regular grid on the map and counted the eye positions that fell within each constituent square (67 m side length) of the grid; points on the edges were assigned randomly to a neighboring square. Next, we transformed these counts to percentages over the total number of eye positions and drew isolines, which connect points of the same probability values. The difference between any two consecutive isolines is the contour interval, and values inside an interval are higher than those outside. The colors of the isolines denote different levels of the contour intervals, with red corresponding to high probability density values, and blue corresponding to low density regions.

**Figure 3:**
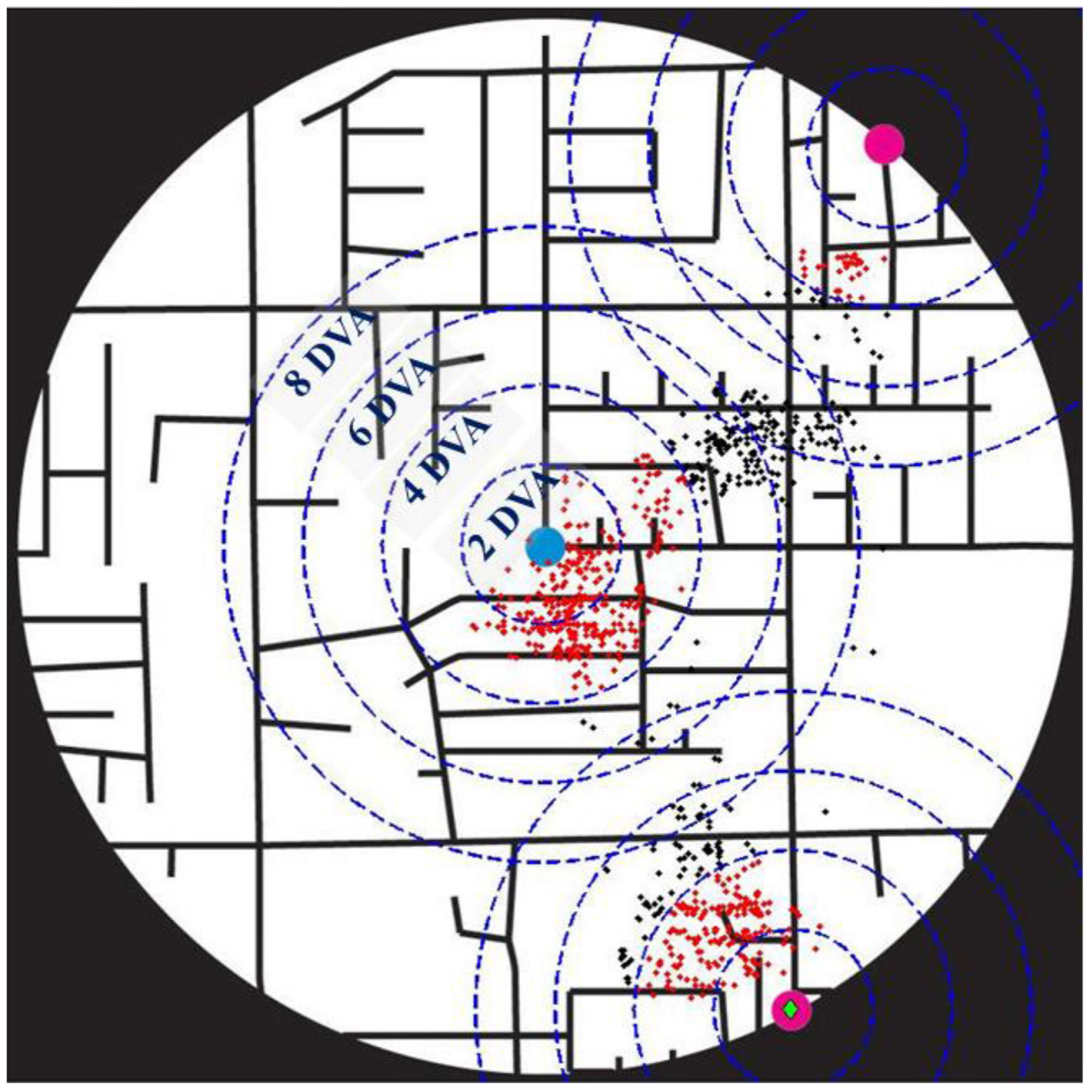
**A single trial illustrating the eye positions (black and red dots) of a subject**. Magenta small circles mark the two targets, a blue small circle denotes the center of the map, and a green diamond marks the selected target. Blue dashed circles correspond to the circular areas of 2, 4, 6, and 8° of visual angle (DVA) radius centered on the two targets and the center of the map, and red dots are the eye positions within 4 DVA-radius around them.

We were interested to quantify the spatial patterns of the eye positions during map exploration, and, particularly, how much of the exploration was spent around the two targets and the center of the map. For that purpose, we calculated (across trials) the mean density (i.e., the mean percentage) of eye positions around the targets and the center of the stimulus, within circular areas of 2, 4, 6, and 8° of visual angle (DVA) radius centered on these locations. Figure 3 displays an example from a single trial, illustrating the eye positions of a subject, and the circular areas of 2, 4, 6, and 8 DVA-radius centered on the two targets and the center of the map.

Finally, to better assess whether eye positions were more densely distributed around the selected targets vs. the non-selected ones, and, hence, to test the hypothesis that subjects spent more time exploring the chosen targets, we computed the mean relative density as the ratio of the frequencies of eye positions within a 4 DVA-radius circle centered on the selected and non-selected targets, to the sum of the number of eye positions within these two circular areas (i.e., around the chosen and non-chosen targets).

#### Temporal analysis of eye positions

**Figure 4:**
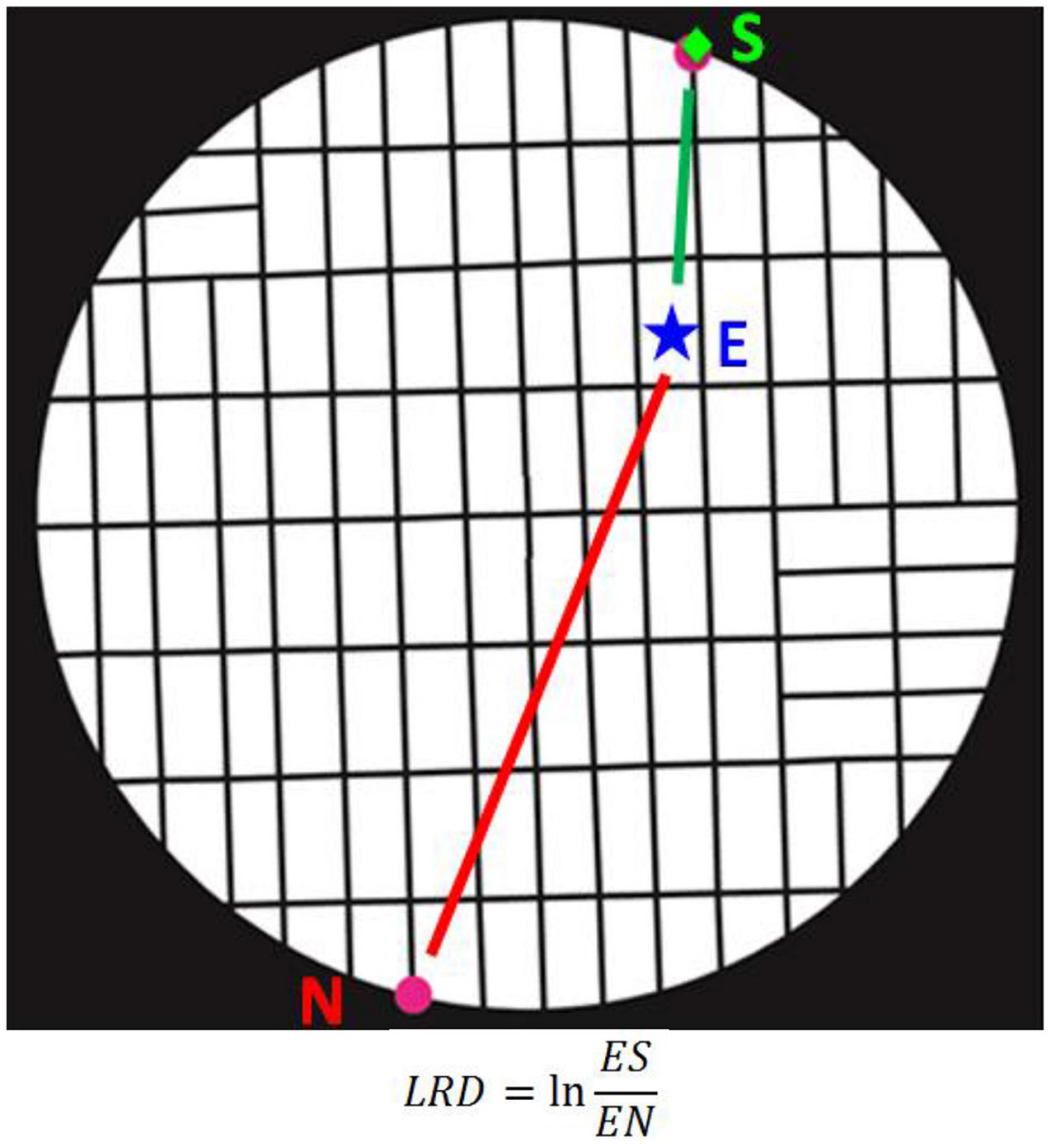
**Illustration of calculation of the LogRatioDistance (LRD) measure**. S, selected target; N, non-selected target; E, current eye position. (See text for details).

To study the temporal evolution of the decision process, we computed the natural logarithm of the ratio of the Euclidean distance between the instantaneous eye positions at time *t* (*eye_X,t_*, *eye_Y,t_*) and the selected target (*Choice_X_*, *Choice_Y_*), to their Euclidean distance to the non-selected target (*NOTChoice_X_*, *NOTChoice_Y_*).

(1) $$\begin{array}{l} \text{LogRatioDistance}\left(t\right)=LRD\\ =\text{ln}\frac{\sqrt{{\left(eye_{X,t}-Choice_{X}\right)}^{2}+{\left(eye_{Y,t}-Choice_{Y}\right)}^{2}}}{\sqrt{{\left(eye_{X,t}-NOTChoice_{X}\right)}^{2}+{\left(eye_{Y,t}-NOTChoice_{Y}\right)}^{2}}}\\ t=1,2,\ldots ,M \end{array}$$

where *M* is the trial length. This measure (see Figure 4 for an illustration) provides an instantaneous metric of the eye trajectories, in terms of how close are the eyes to the selected vs. non-selected target. Negative *LRD* values indicate eye positions closer to the selected target, and vice-versa.

#### Computation of street network distance measures

**Figure 5:**
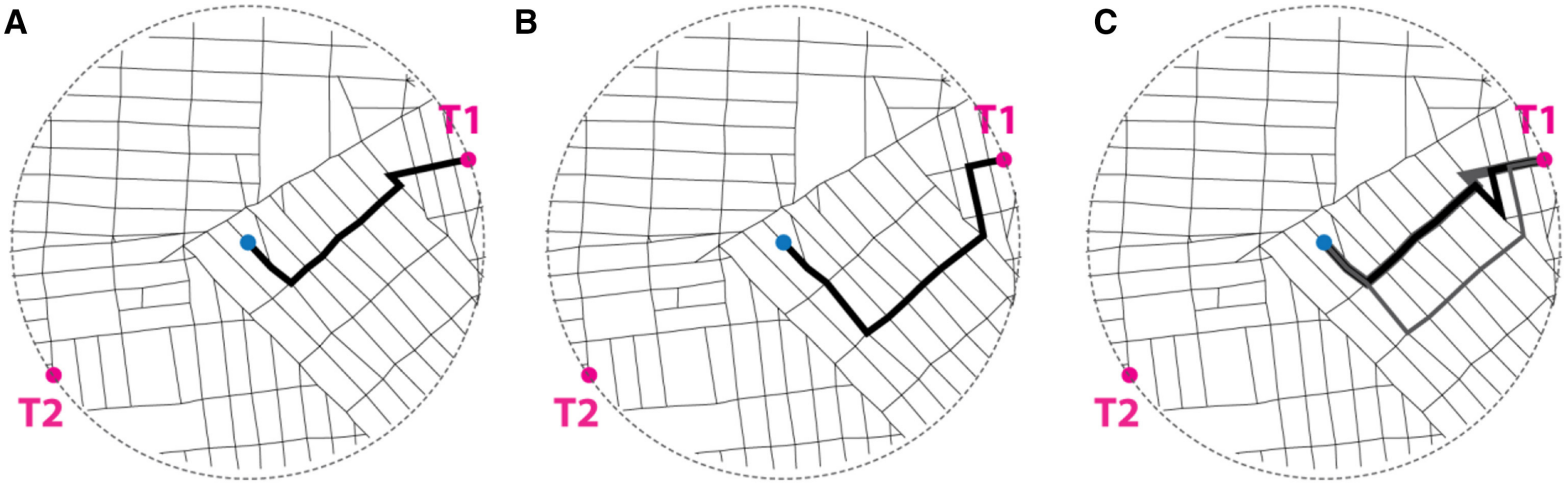
**Illustrative definition of measures of network distance. (A)** *Minimum path length* between the center and T1 is 3381.85 feet (1030.79 meters). In this instance there is a uniquely defined path of such length, shown in thick black line. **(B)** *Minimum path rotation* between the center and T1 is 284.58°. In this instance there is a uniquely defined path of such minimum aggregate rotation, shown in thick black line. Note that it is not the same as the path of minimum length. **(C)** The *minimum street crossing* between the center and T1 is 12. There are at least 6 paths that satisfy this condition. Three of those are shown, in varying line thicknesses and shades, so that they may be distinguished. In this instance, the paths of minimum length and minimum rotation are both included in the set of paths that go through the minimum number of street crossings.

The Cartesian straight-line distance from the center of a stimulus map to a target is half a mile in all stimuli. The actual street network distance varies. To study the spatial properties underlying the choice of the post-office target location, the following measures of street network distance were computed for all targets and stimuli. (1) The shortest available metric distance (i.e., *Minimum path length*): The length of the shortest available path linking the center to each of the targets, measured in feet. (2) The shortest available angular distance (i.e., *Minimum path rotation*): The sum of angles of direction changes along the straightest available path linking the center to each target. (3) The shortest available intersection distance (i.e., *Minimum street crossings along the path*): The number of street intersections crossed along the simplest available path linking the center to the target; for the purpose of this calculation, an intersection is defined as a node with at least three incident road segments, offering at least two choices for movement. It is noted that while in some cases the same individual path minimizes all three measures, in other cases different paths might be involved. For example, the shortest and straightest path may cross more intersections than a less direct path. In our analysis of the stimuli maps, all the paths available are evaluated according to each measure of distance independently. The first measure was computed using Network Analyst on ArcGIS10. The last two measures were computed using Spatialist-lines, a set of specialized routines for spatial analysis developed at Georgia Tech in collaboration with Perkins + Will. These routines are implemented using Grasshopper, a language for algorithmic modeling associated with design modeling software Rhinoceros. Figure 5 illustrates the definition of the 3 measures used in the analysis.

#### Statistical analysis

Data were analyzed using standard statistical methods and either the ISPSS IBM statistical Package (version 21) or MATLAB® (version R2013b).

## Results

### Decision time

**Figure 6:**
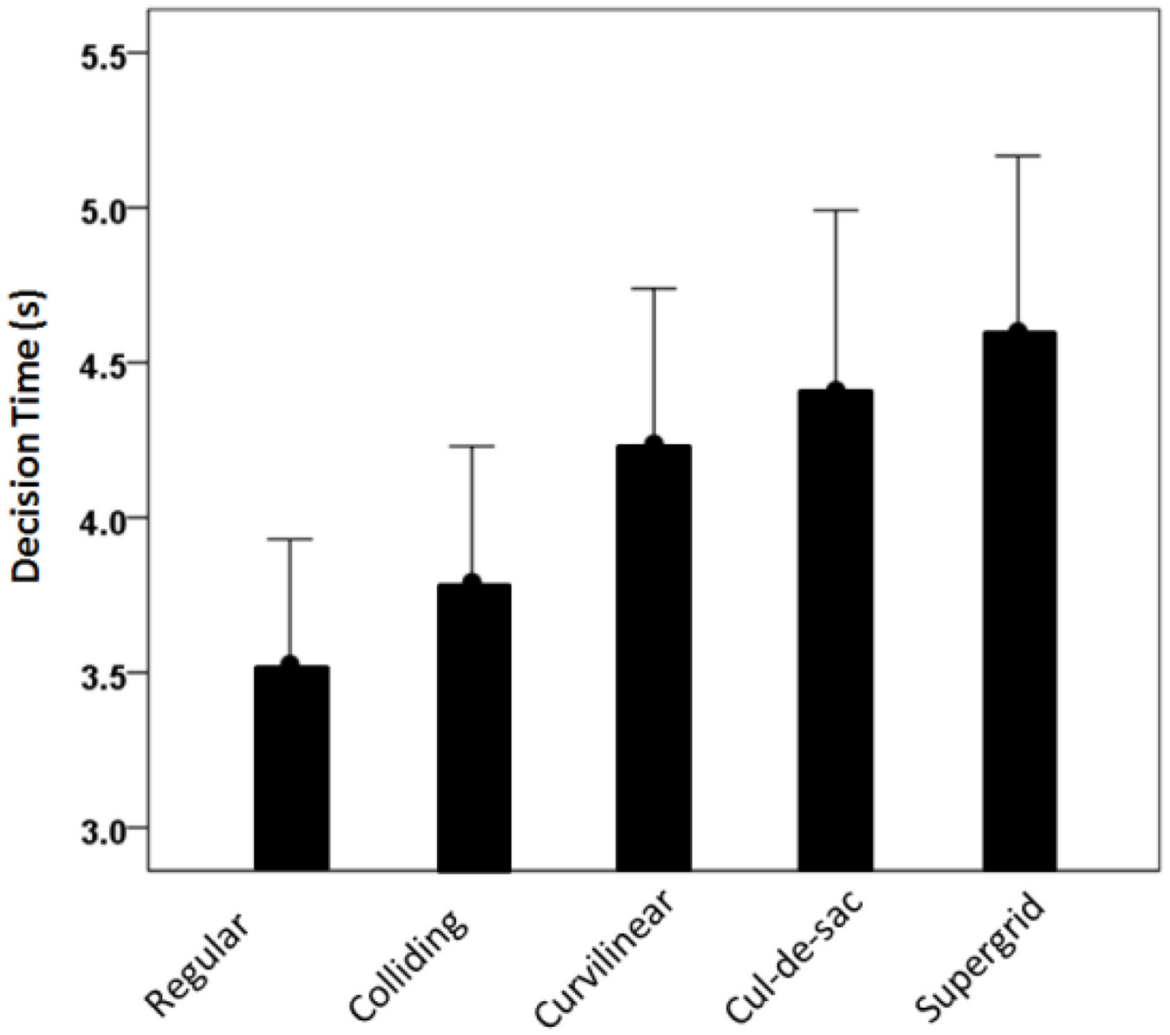
**Decision time (mean ± SEM) for different street network types**.

Subjects decided on the post office choice after 4.113 ± 0.225 s (mean ± SEM, *N* = 236 valid trials). We performed an analysis of variance (ANCOVA) to assess the effects of street network type on decision time, where the decision time was the dependent variable, the angle formed by the two radii of the targets was a covariate, and the subject was a random factor. We found a statistically significant effect of the street network type (*P* = 0.016, *F*-test) (Figure 6). The effect of the target angle was marginal (*P* = 0.051), the effect of the subject random factor highly significant (*P* < 0.001) but without a significant effect of the network × subject interaction term (*P* = 0.747).

### Spatial characteristics of eye positions

**Figure 7:**
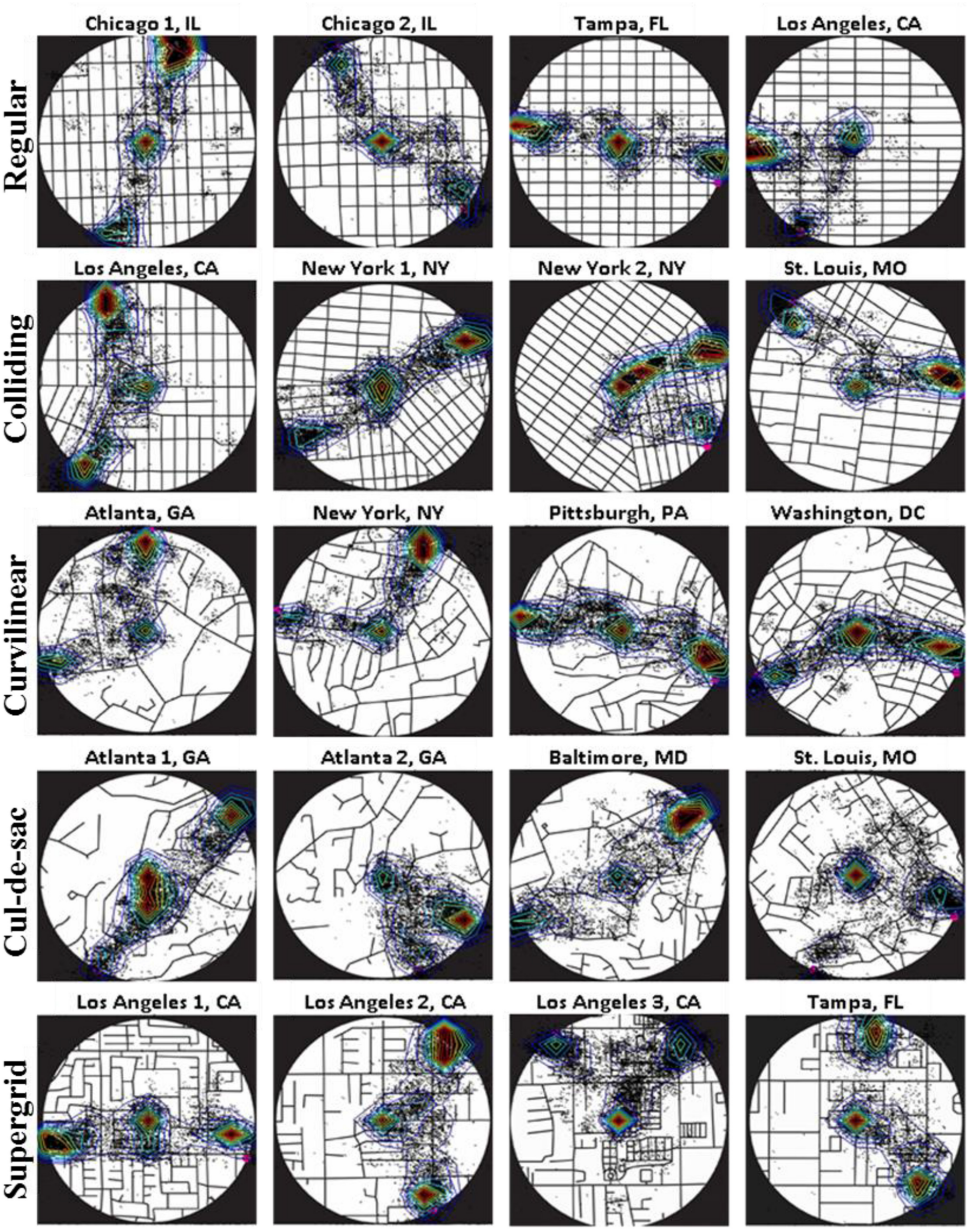
**Superimposed eye positions (black dots) on each map of all 12 subjects, and the corresponding isolines illustrating the probability density of the eye positions**. Isoline colors describe different levels (0–1) of the contour intervals, with red corresponding to high probability density values, and blue corresponding to low density values.

Figure 7 depicts the superimposed eye positions of all subjects on each map and the corresponding contour plots that describe the probability density of the eye positions by isolines (see Methods above for more details). It can be seen that subjects explored the map mostly around the center of the map and the two alternative locations (see the contour intervals within the red isolines in Figure 7). This pattern of eye positions was consistent across all maps irrespectively of street network type and target configuration.

**Figure 8:**
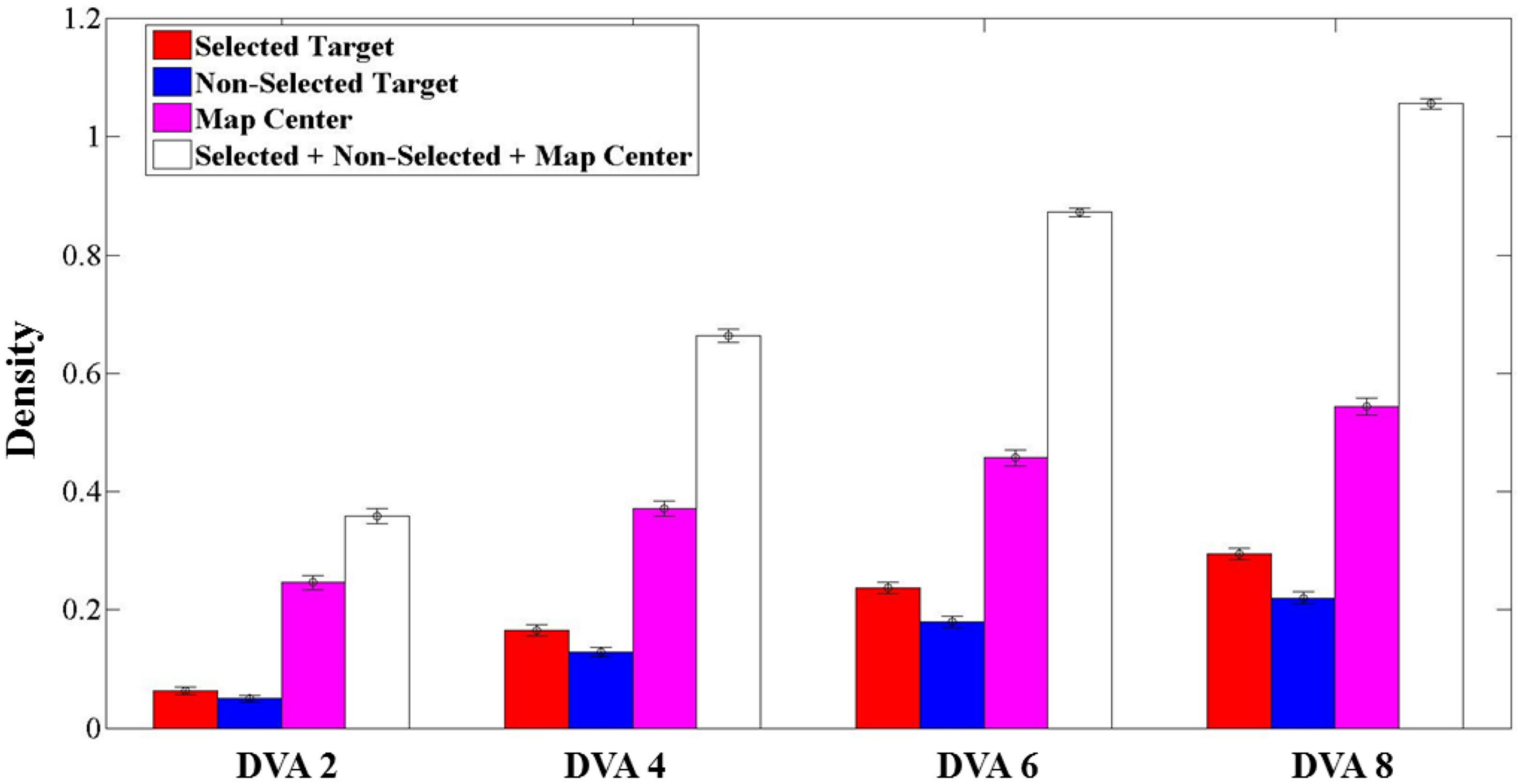
**Average density (mean ± SEM, *N* = 236 trials) for selected targets (red), non-selected targets (blue), center of the map (magenta), and the combination of them (white), calculated in the circular area of 2, 4, 6, and 8° of visual angle (DVA) centered on each one of them**.

To further quantify the spatial pattern of eye positions, we calculated the mean density of eye positions across trials, within circular areas of 2, 4, 6, and 8 DVA-radius centered on the selected target, the non-selected target, and the center of the map (Figure 8). We found that more than 50% of the mean density at 4 DVA-radius, (0.664 ± 0.011, mean ± SEM), was attributed to both targets and the center of the stimulus (see white bars of Figure 8). This means that most eye positions fell within a circular area of 4 DVA-radius around these locations, suggesting that subjects were exploring only a relatively small region around the targets and the center of the map. Since such circular areas of 4 DVA-radius captured most visual fixations, we used 4 DVA-radius areas for subsequent quantitative analyses.

### Time spent in exploring the alternative options and choice bias

**Figure 9:**
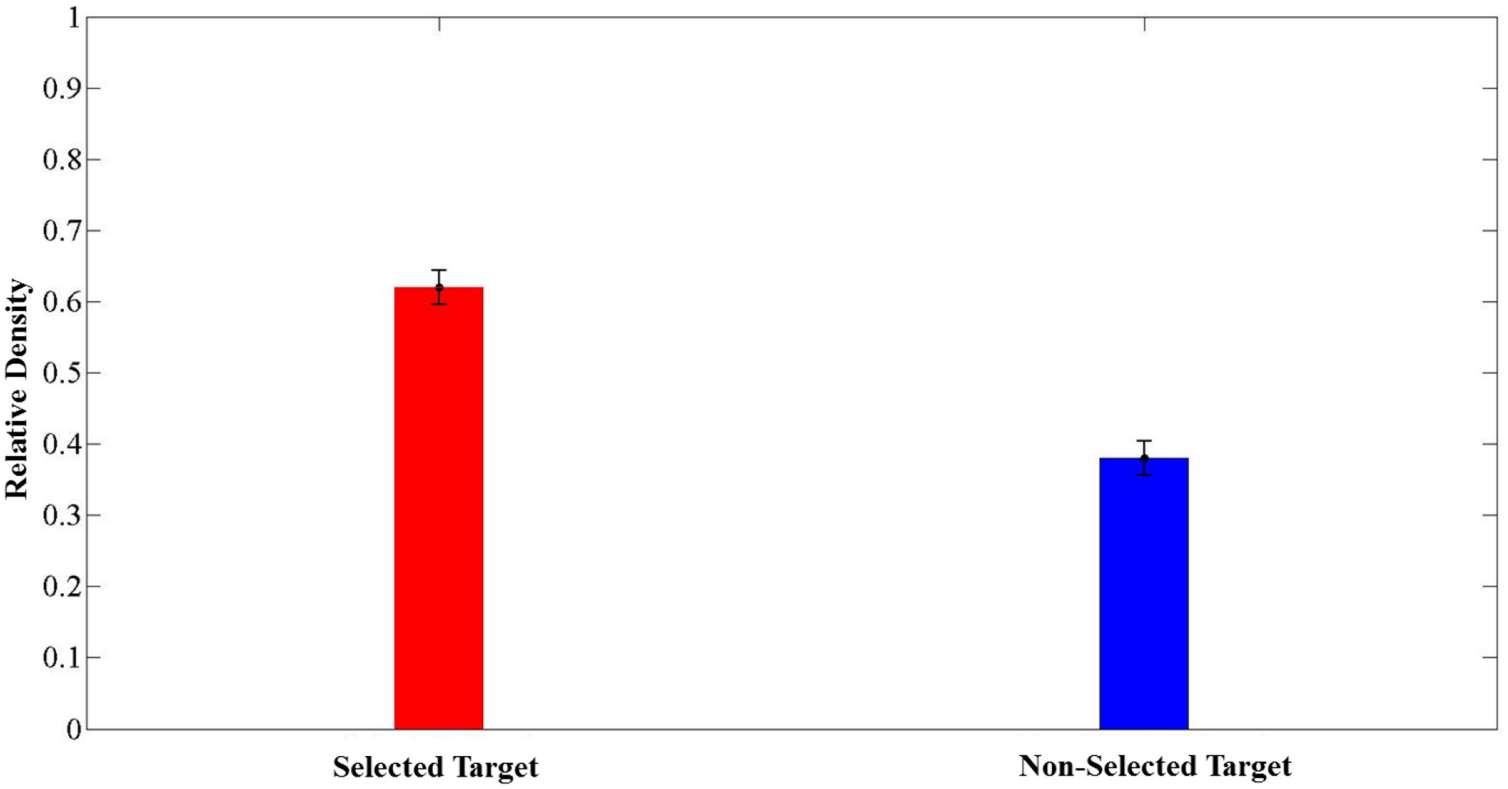
**Average relative density for selected and non-selected targets in the circular area of 4° of visual angle centered on each target (mean ± SEM, *N* = 209 trials)**. Note that the relative density was computed as the ratio of the frequencies of eye positions within a 4 DVA-radius circle centered on the selected and the non-selected targets, to the sum of the number of eye positions within these two circular areas.

We were also interested in investigating whether the choice of an option was related to the time that subjects spent exploring the area around it. Our initial hypothesis was that subjects spent more time looking at the selected than the non-selected targets. To test this hypothesis, we calculated the mean relative density (see Methods) of eye positions within the 4 DVA-radius areas around the selected and non-selected targets across trials. We found that subjects spent on average more time exploring the region around the selected location than the non-selected one (Figure 9). In addition, there was a significant association between the selected target and the target with the higher relative density within 4 DVA-radius around that target (χ^2^ = 4.207, *P* = 0.03).

### Comparison of alternative options as a function of time

**Figure 10:**
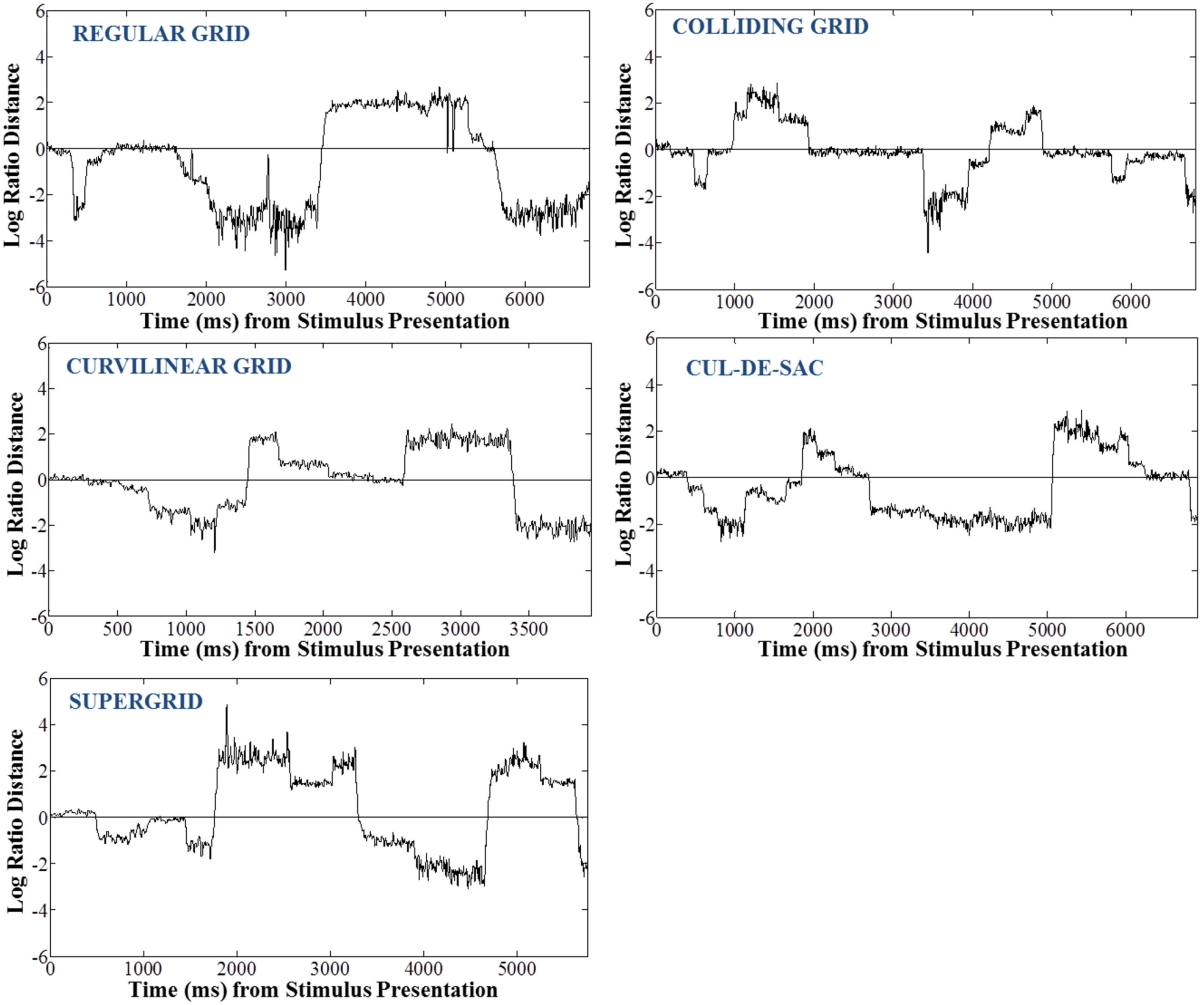
**Example trials of 5 subjects while exploring different types of maps—one for each street network type—illustrating the log-ratio distance of instantaneous eye positions to selected target over the non-selected target**. Negative values of the log-ratio distance correspond to eye positions closer to the selected target, and vice-versa.

**Figure 11:**
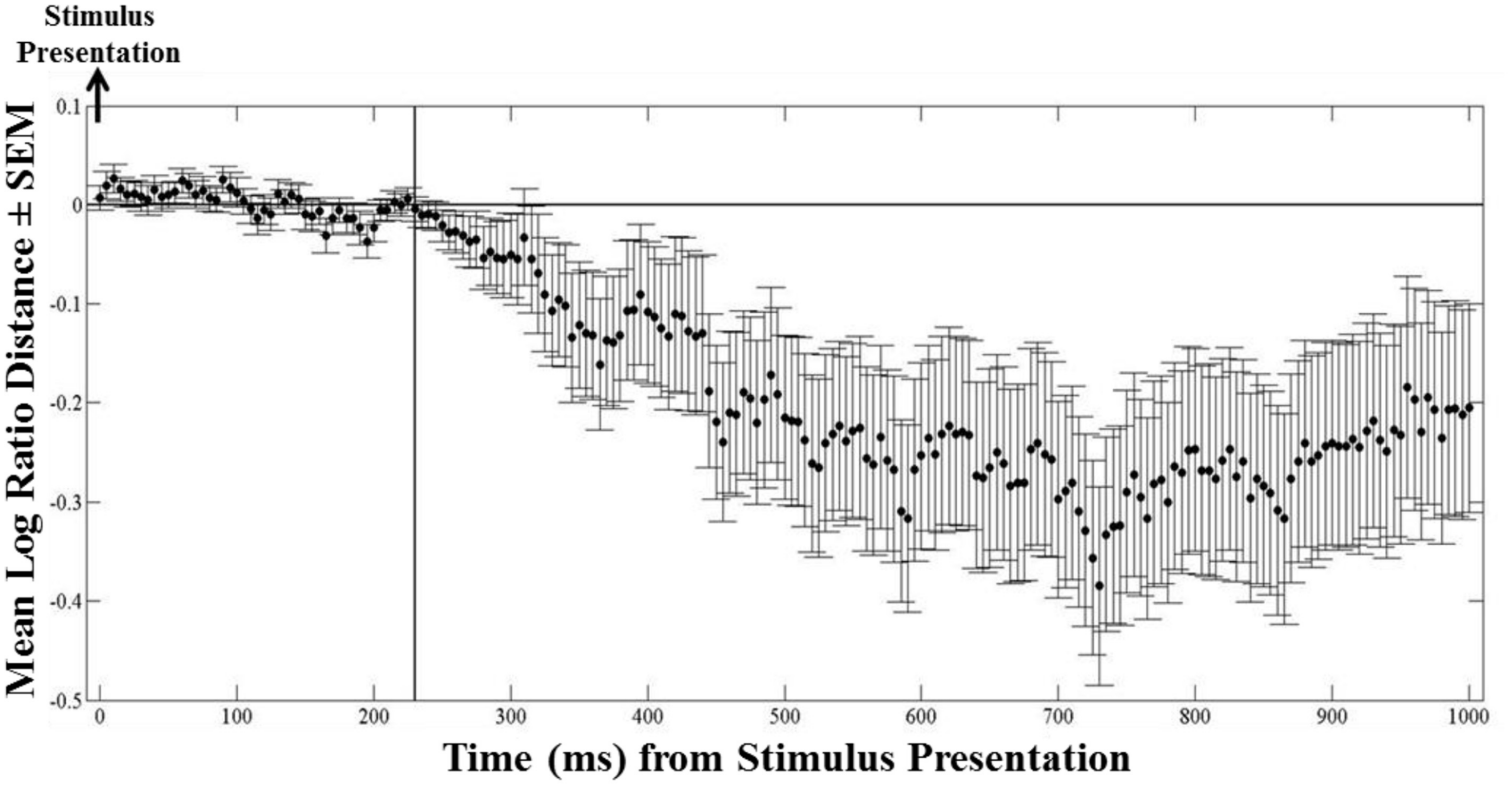
**Mean logarithmic ratio (mean ± SEM, *N* = 236 trials) of the Euclidean distance of the ongoing eye position to the selected target to the Euclidean distance of the eye position to the non-selected target, for 1 s after stimulus presentation**. Trials are aligned to stimulus presentation. Notice that as early as 230 ms after stimulus onset occurred the initial fixation target bias—values of the mean *LRD* become negative (i.e., eyes are getting closer to the selected target).

**Figure 12:**
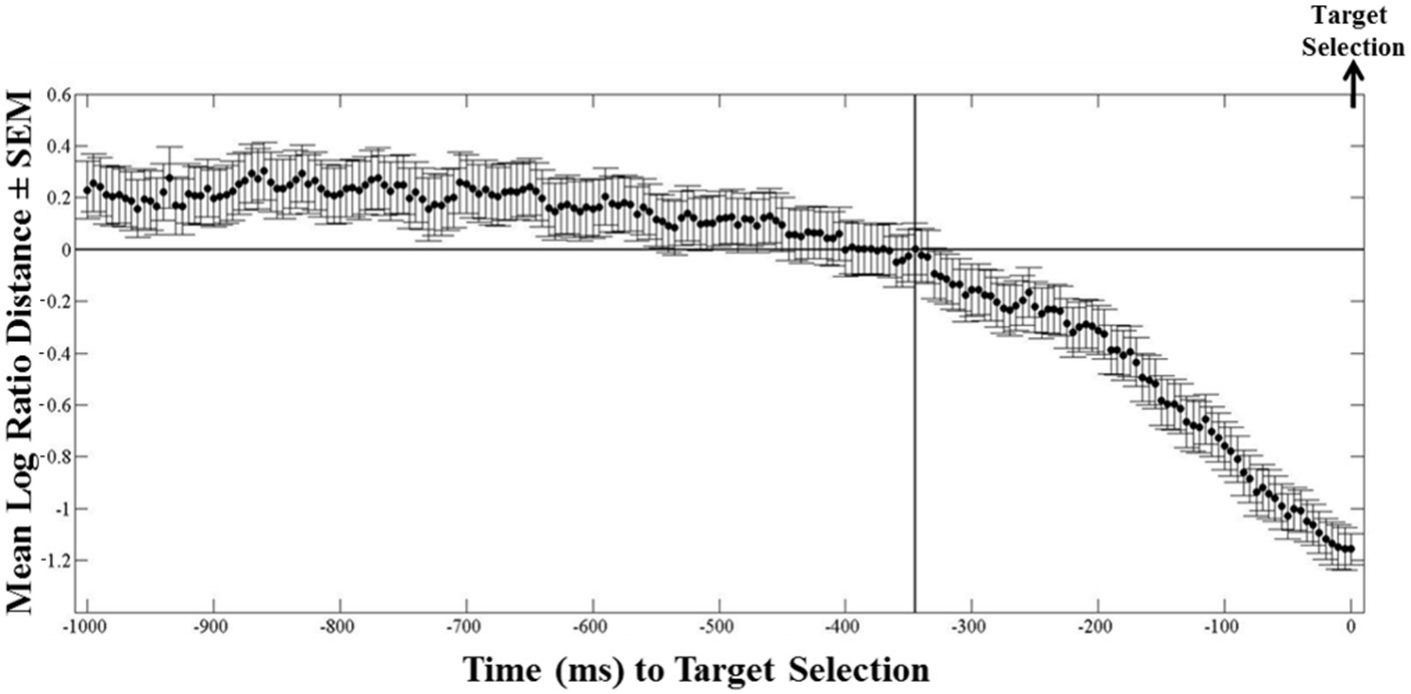
**Mean logarithmic ratio (mean ± SEM, *N* = 236 trials) of the Euclidean distance between the ongoing eye position and the selected target, to the distance between the eye position and the non-selected target, for 1 s before target selection**. Trials are aligned to target selection. 345 ms before the selection of the target, mean *LRD* values become negative (i.e., subjects on average started moving their eyes close to the selected target), indicating the last fixation target bias.

**Figure 13:**
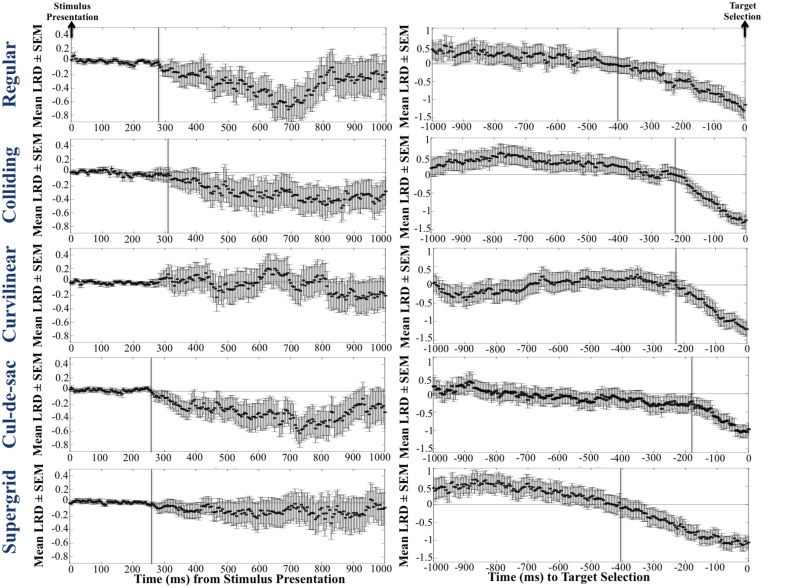
**Mean LRD for each street network type (each row corresponds to a single grid), for 1 s after the map onset (first column), and 1 s before the target selection (second column)**. Note that in the first column, trials are aligned to map presentation, whereas in the second column trials are aligned to target selection.

We used the instantaneous *LRD* (see Methods) to monitor eye position with respect to its proximity to the selected (or non-selected) target. Characteristic *LRD* time courses from different subjects, trials and maps, are shown in Figure 10. It can be seen that subjects spent chunks of time closer or farther away from the ultimately selected target, as indicated by the negative and positive *LRD* values, respectively. However, we found that, on the average, subjects fixated points nearer the selected (vs. the non-selected) target both at the beginning and the end of the trial. We quantified this observation by aligning all the trials, in different analyses, either (a) to the onset of map presentation, or (b) to the moment of target selection (mouse click) and computing the average instantaneous *LRD*. The results are shown in Figures 11, 12, respectively. It can be seen that subjects showed a systematic bias toward the target-to-be-selected, beginning approximately 230 ms after map onset (Figure 11), and 345 ms before target selection (Figure 12). We found similar results when computing the average instantaneous *LRD* for each street network type (Figure 13).

### Evaluation of alternative options based on network distance measures

**Figure 14:**
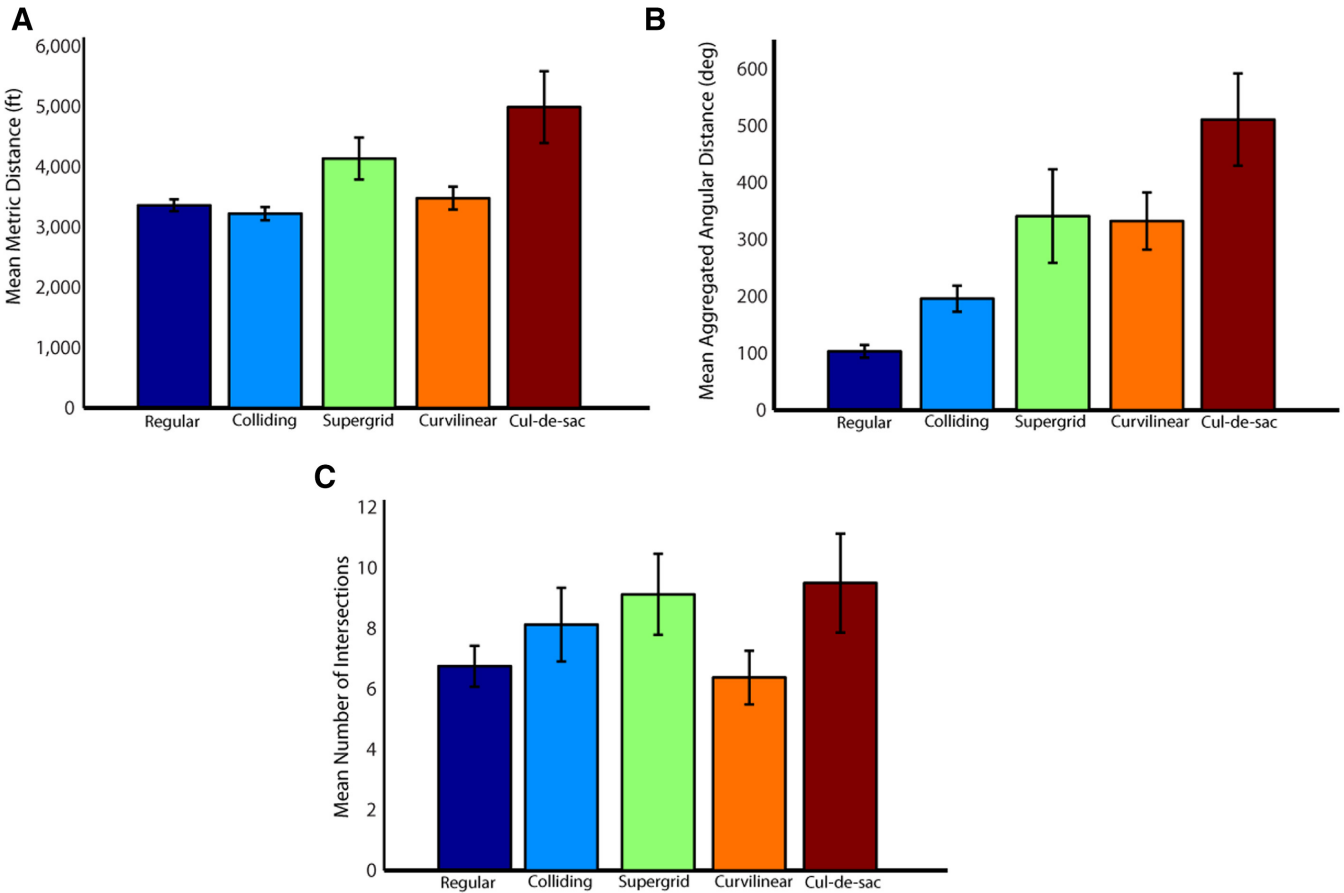
**Mean network distance measures (mean ± SEM) across all target locations for each street network type. (A)** Mean shortest available metric distance from the center to the selected and non- selected targets, measured in feet. **(B)** Mean shortest available angular distance, measured by the sum of all angles of direction change needed to move from the center to the selected and non-selected targets. **(C)** Mean shortest available intersections distance, measured by the minimum number of intervening intersections needed to move from the center to the selected and non-selected targets.

**Figure 15:**
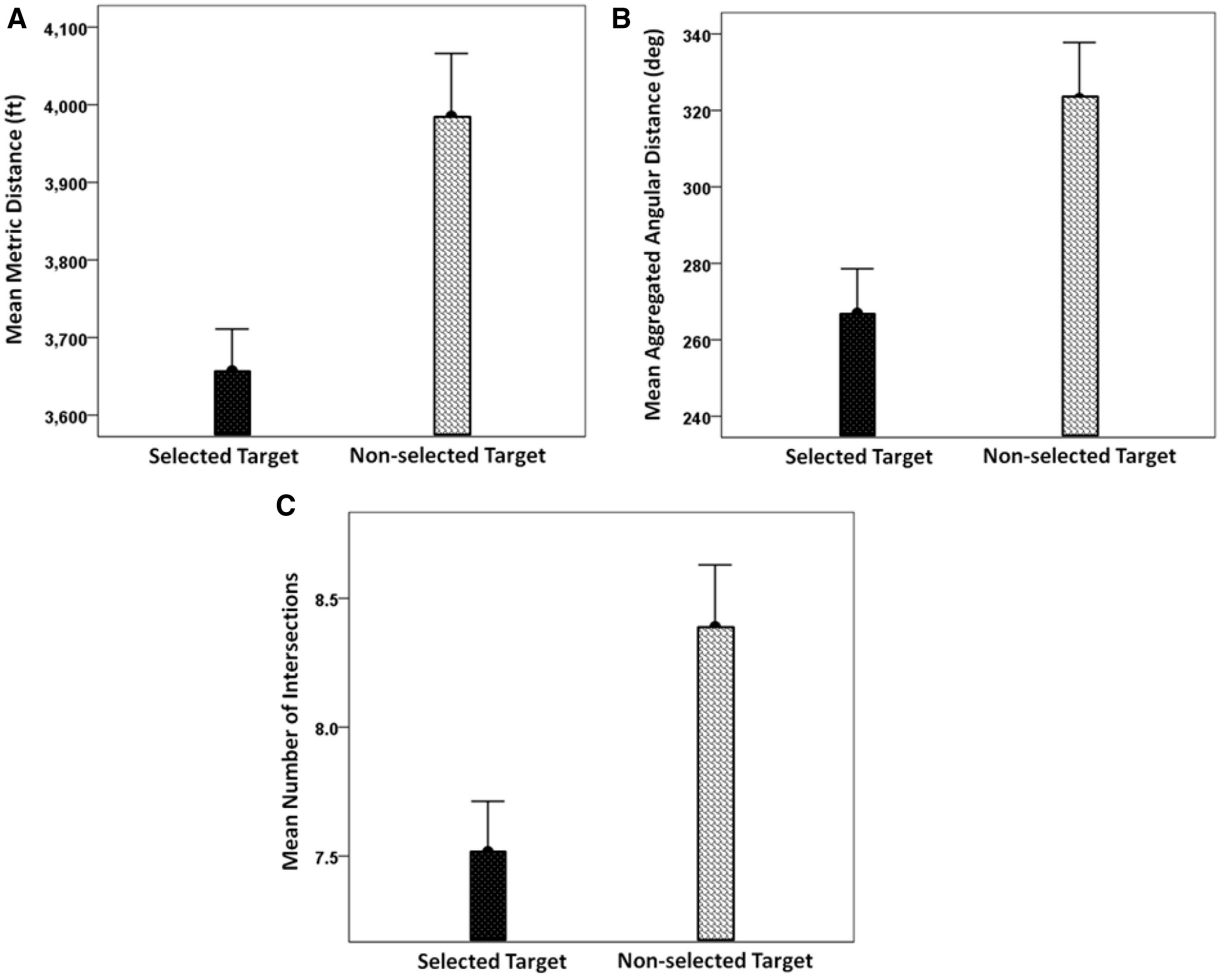
**(A)** Mean shortest available metric distance from the center to the selected and non-selected targets, measured in feet. **(B)** Mean shortest available angular distance, measured by the sum of all angles of direction change needed to move from the center to the selected and non-selected targets. **(C)** Mean shortest available intersections distance, measured by the minimum number of intervening intersections needed to move from the center to the selected and non-selected targets. (mean ± SEM, *N* = 209 trials).

We evaluated the alternative options based on 3 network distance measures namely (i) minimum path length, (ii) minimum path rotation, and (iii) minimum street crossings along the path (see Methods Section for more details). The mean and the standard error of these 3 measures across all target locations for each street network type is shown in Figure 14. Additionally, Figure 15 depicts the results of the space syntax map path analysis for the three network distance measures. It can be seen that all of them were substantially larger for the non-selected target (*P* < 0.001, paired *t*-test).

## Discussion

The ability to explore novel environments and make spatial decisions, such as selecting a place to live or walking toward a landmark, is a fundamental and highly evolved behavior that requires the coordination of cognitive functions. In the recent years, significant progress has been made in understanding the cognitive mechanisms of exploration and decision-making. Many studies have investigated how people and animals explore and navigate in novel environments (Spiers and Maguire, 2006, 2007), whereas others have focused on understanding how they select between alternative options that have economic consequences (Wunderlich et al., 2010; Rangel and Clithero, 2013; Towal et al., 2013). Despite the important findings from these studies, little is known about the strategies that people and animals adopt when they are faced with both problems, i.e., exploring novel environments to make decisions. To address this question, we designed an experiment to study how people make spatial decisions while exploring realistic environments. We used a variety of real maps of various U.S cities with different street network layouts, and marked on each map two potential locations for a hypothetical post office. We asked 12 subjects to choose one of the two alternative targets to post their mail, by moving a mouse cursor from the center of the map and clicking at the selected point. On the average, subjects decided on a target fastest when presented with a regular street network type and slowest when presented with a supergrid. Interestingly, there was an orderly increase of the decision time, with respect to the street network type, such that colliding, curvilinear, cul-de-sac and supergrid types had progressively longer decision times (Figure 6). This probably reflects the simplicity of the regular grid, the presence of curved streets, and the highest complexity in the supergrid. However, it could be argued that the effects of the street network on the decision type may not necessarily be associated with the realistic features of the city maps. Instead, it may be related to low level visual salience. Particularly, the decision time may increase with the visual complexity of the maps. However, this view does not explain why the 3 measures of network distances were significantly different for the selected than the non-selected targets, and why the subjects were repeatedly looking between the two options before making a decision. Overall, it is possible that the complexity of the map influences the decision time, but our findings suggest that people take into account the geometric characteristics of the maps to evaluate the alternative options and make decisions.

### Center → target path

Next, we explored the possibility that a target was selected based on the navigational properties of the path from the center to the target. Specifically, we hypothesized that, as subjects first fixate at the center, they might use that as a vantage point from which to get to a location. In that case, it makes sense to suppose that the easiness of reaching a destination might play a role in deciding which of the two targets to choose. Now, the degree of “easiness” of the path from the center to a target can be evaluated within the context of space syntax. In a sense, “easiness” means straight streets with few turns and few stops at intersections. We used space syntax analysis to quantify these aspects of path navigation by computing quantitative measures of metric distance, angular distance and intersections for each path from the center to a target. Indeed, we found that all these three distance measures were significantly smaller for the selected targets than the non-selected targets (Figure 15). This suggests that subjects probably mentally navigated the two paths and ultimately “went” to the target with the “least-resistance” path. It should be mentioned that the space measures above have been traditionally used in the literature on environmental perception, cognition and navigation in urban environments (Lee, 1970; Sadalla and Lorin, 1980; Sadalla and Magel, 1980; Sadalla and Montello, 1989; Montello, 1991; Bailenson et al., 1998, 2000; Conroy-Dalton, 2003; Jansen-Osmann and Wiedenbauer, 2004).

### Decision-making strategy

We used eye position to investigate and evaluate possible strategies underlying map exploration and decision making. Indeed, monitoring subjects' eye position revealed that people developed highly stereotyped strategies for evaluating and comparing the two potential locations. Results showed that people followed a restricted exploration delimited by the center and the two targets. Specifically, subjects continuously explored the areas around the two targets, and the space between center and target (in a band fashion) before making a decision. This behavior is in accord with the hypothesis that subjects evaluated space syntax network characteristics, as discussed above.

Another interesting finding was that the eye fixation biased the decision toward the location that was being fixated most of time. This effect has been also described in value-based decision studies (Pieters and Warlop, 1999; Shimojo et al., 2003; Simion and Shimojo, 2006, 2007; Krajbich et al., 2010; Krajbich and Rangel, 2011; Towal et al., 2013). According to these studies, the longer you spend looking at a good you like, the higher the probability to select that good than the alternative options. For instance, when you buy a car, you may spend a lot of time test-driving and reviewing the car specifications before buying it. Recent computational theories developed to understand how people decide between competing options (Krajbich et al., 2010; Krajbich and Rangel, 2011). According to these studies, when we are faced with multiple alternative options, the brain assigns a relative decision variable to each of these alternatives and implements a comparison process by repeatedly looking at them. The relative decision variable assigned to a good is positively correlated with the time that subjects fixate on it (Krajbich et al., 2010; Krajbich and Rangel, 2011; Towal et al., 2013). Hence, the probability to select a good increases the longer the good is fixated on. According to these findings, we may also assume that when subjects are spending more time exploring the area around, and the path toward, a target, the value of this location (and associated path) increases, and, consequently, this location becomes more likely to be selected as the post office location.

Besides the similarities between our results with findings from value-based decision studies, we found that our results are not consistent with the gaze cascade effect hypothesis reported in economic choices (Shimojo et al., 2003). According to this hypothesis, when people have to select between two targets, the gaze is initially distributed evenly between the two targets; then, it is gradually shifted toward the target that it is eventually selected. Counter to this hypothesis, we found that subjects had a strong bias to select the location they firstly explored after the map onset. This bias appeared at around 230 ms after the presentation of the stimulus. Similar findings have also been reported in many studies involving choices between multiple goods, which showed that the probability of first-seen item is chosen increases with the duration of the first fixation (Krajbich et al., 2010).

Additionally, a recent study explored the neural basis of choice bias using magnetoencephalography (MEG) in a value-based decision task and found that MEG signal deviations from biased decisions occurred as early as 250–750 ms following the stimulus onset (Hedgecock et al., 2010). The presence of an early bias for upcoming decisions raises the issue as to why subjects take seconds to decide instead of choosing right away. A reasonable hypothesis is that this early bias might not, in fact, carry sufficient weight to force a decision, for which more exploration time is needed. This hypothesis remains to be tested rigorously in additional experiments.

### Conflict of interest statement

The authors declare that the research was conducted in the absence of any commercial or financial relationships that could be construed as a potential conflict of interest.
